# Detecting rater bias using a person-fit statistic: a Monte Carlo simulation study

**DOI:** 10.1007/s40037-017-0391-8

**Published:** 2018-01-02

**Authors:** André-Sébastien Aubin, Christina St-Onge, Jean-Sébastien Renaud

**Affiliations:** 1 Université de Sherbrooke, Sherbrooke, Québec Canada; 2 Université Laval, Québec, Québec Canada

**Keywords:** Rater-based assessment, Person-fit statistics, Detection rates

## Abstract

**Introduction:**

With the *Standards* voicing concern for the appropriateness of response processes, we need to explore strategies that would allow us to identify inappropriate rater response processes. Although certain statistics can be used to help detect rater bias, their use is complicated by either a lack of data about their actual power to detect rater bias or the difficulty related to their application in the context of health professions education. This exploratory study aimed to establish the worthiness of pursuing the use of *l*_*z*_ to detect rater bias.

**Methods:**

We conducted a Monte Carlo simulation study to investigate the power of a specific detection statistic, that is: the standardized likelihood *l*_*z*_ person-fit statistics (PFS). Our primary outcome was the detection rate of biased raters, namely: raters whom we manipulated into being either stringent (giving lower scores) or lenient (giving higher scores), using the *l*_*z*_ statistic while controlling for the number of biased raters in a sample (6 levels) and the rate of bias per rater (6 levels).

**Results:**

Overall, stringent raters (*M* = 0.84, *SD* = 0.23) were easier to detect than lenient raters (*M* = 0.31, *SD* = 0.28). More biased raters were easier to detect then less biased raters (60% bias: 62, *SD* = 0.37; 10% bias: 43, *SD* = 0.36).

**Discussion:**

The PFS *l*_*z*_ seems to offer an interesting potential to identify biased raters. We observed detection rates as high as 90% for stringent raters, for whom we manipulated more than half their checklist. Although we observed very interesting results, we cannot generalize these results to the use of PFS with estimated item/station parameters or real data. Such studies should be conducted to assess the feasibility of using PFS to identify rater bias.

## What this paper adds

In a Monte Carlo simulation study we applied the *l*_*z*_ person-fit statistic to detect biased raters in the context of an objective structured clinical examination (OSCE). The study was conducted to overcome limits of previous studies on rater detection (e. g., lack of empirical support of their power, or issues of feasibility). We observed that the *l*_*z*_ statistic can detect up to 90% of stringent raters, that is: raters who score examinees’ performance lower than expected. Our results suggest that the use of a detection statistic, namely *l*_*z*_, could become an interesting tool in the context of in-house rater-based assessment validation process.

## Introduction

The increased popularity of competency-based education has resulted in many changes to assessment [1, 2]. More specifically, there is increased reliance on performance-based assessment and, consequently, on raters to assess examinees [2–5]. Gauthier et al. [6] have summarized the literature on rater cognition, and have eloquently illustrated its complexity and multidimensionality in a three-phase, nine-mechanism model. While many authors have supported the subjective nature of rater-based assessment (RBA) [2, 7–9], what can be gleaned from the broader literature on RBA is that its use, although inevitable, is often criticized based on the growing amount of evidence on rater bias [10–17]. In other words, although subjectivity, and thus differences between raters can be seen by some authors as an added value to performance-based assessment, this subjectivity is categorized as a source of measurement error by other authors [18].

Research has shown that errors in rater judgment can be linked to biases such as the contrast effect, the confirmation bias, the self-fulfilling prophecy, or the assimilation effect [13–15]. Knowledge of an examinee’s prior performances, values, work habits, and demographics is also known to influence raters’ judgement [11, 19–22]. These rater biases may yield examinee scores that do not strictly reflect their performance [23].

The literature on raters indicates differing results on how much variance rater biases contribute to score variability. In fact, studies have shown raters to contribute as little as 9% to the observed score variability [24] or as much as 44% [25]. Most studies in health professions education (HPE) that have attempted to quantify rater variance used either generalizability theory or the many-facet Rasch model (MFRM), and they report rater variances between 12 and 22% [26–28]. Observed differences in rater variances could be due, at least in part, to the nature of the examination. Some studies were conducted in a high-stakes setting, such as Bartman et al. [29], who investigated a national licensure examination over a 3-year period and observed lower rater variance, while other studies, such as Harasym et al. [25], were conducted at the level of an in-house performance-based assessment and observed an even greater rater variance. Researchers have also investigated the downstream effect of observed rater variance on assessment outcome, namely pass-fail decisions. Again, there is some variability in the observed results, but studies have shown that rater variance could lead to changes in decisions (pass to fail, or fail to pass) for 4 to 11% of examinees. The magnitude of changes in assessment outcomes seems to be strongly related to the relative importance of rater variance. For example, Harasym et al. [25] observed the greatest rater variance (44%) and also the most change in assessment outcome when controlling for rater variability. Consequently, this would reduce the validity of the score interpretation for the assessment [23].

Statistical strategies have been used in HPE to identify biased or extreme raters. More specifically, we will discuss four different statistical strategies. Bartman et al*. *[29] proposed a simple three-step statistical model to detect extreme *stringent and lenient raters* based on rater deviation compared with the station mean. They used this modelling in the context of a 12-station, high-stakes objective structured clinical examination (OSCE). They reported that fewer than 1% of raters were identified as biased raters (in terms of extreme ratings). While they offer one of the more accessible strategies for extreme rater identification, they conducted a case study in a naturalistic setting and, as such, the specificity and sensitivity of their proposed strategy are impossible to establish.

The MFRM can be used to identify, and then statistically correct for, biased raters (i.e., identify outlier raters and subsequently standardize their score to the group mean; [25, 28, 30]). The use of this model generally requires a large sample. More importantly, the use of MFRM to identify extreme raters requires a setting in which there is adequate linking that allows the researcher to estimate rater severity and station difficulty independent of examinee ability levels [28, 30]. In other words, to use MFRM, we need to be able to identify which factors contribute to the examinee score variation, for example, rater severity or station difficulty. When raters are nested within a station (i. e., assigned to a given station) and the stations measure different contents (and examinee ability varies from station to station and cannot be used to set a baseline comparison), proper data linkage for rater severity estimation is almost impossible to achieve.

Boulet et al. [26]. used the MuD statistic, which is ‘the average difference in ratings between the standardized patient performing the case and the observer, averaged over the chosen level of interest’ (p. 40). Similar to Bartman et al. [29], the computational aspect of the MuD statistic is somewhat accessible; however, as with the application and use of MFRM, the MuD statistic requires assessment of examinees within a given station by two different raters, which is not the norm in the context of in-house performance-based assessments.

Finally, Raymond and Viswesvaran [31] focused their efforts on correcting the assessment scores using statistical tools based on distance of examinee to the mean. More specifically, they used the ordinary least squares and the weighted least squares methods. With these techniques, the pass/fail status of 5.2 to 10.5% of examinees can be changed. However, it was impossible to determine the precise number of biased raters. In summary, results from the literature do not allow us to establish the specificity and sensitivity of the proposed statistics, as all studies were conducted in a naturalistic setting.

Looking at the broader literature on fit detection, there seems to be a family of statistics, i. e. person-fit statistics (PFS), which has been shown to be robust in its initial context (detecting aberrant examinees) and has yet to be investigated in the context of detecting biased raters. PFS are traditionally used to detect aberrant examinees, namely: examinees who have unlikely answer patterns according to their ability level and the estimated item parameters [32]. There are more than 50 different PFS [33]. However, they are similar in that they aim to estimate the probability that an examinee with a given ability level will produce a specific answer pattern. In other words, they are intended to assess the fit between an examinee’s overall score and the answers given to the questions. Different examinee aberrance types can seemingly be detected using PFS, namely cheating, guessing, undefined spuriously low or spuriously high scores, and even extremely creative examinees [34, 35]. This is of interest in the context of RBA, in which different biases may manifest themselves differently in the raw data. Thus, PFS, akin to the MFRM, could be used to detect various biases in a sample without the caveat, however, of multiple raters per examinee within a station. When using banked OSCE stations (with known item properties), PFS do not require large samples.

The* standardized likelihood l*_*z*_ index is one of the most popular PFS [33, 36, 37] because it works as well as—and often better than—several other PFS [38]. More importantly, the *l*_*z*_ PFS seems amenable to the detection of rater bias without modification. The *l*_*z*_ PFS is used to establish the likelihood of a given examinee answer pattern, given item parameters (e. g., difficulty and discrimination), and examinee ability estimates. In other words, *l*_*z*_ establishes if an answer pattern is probable or improbable given the examinee’s ability level and the exam specificity. Translated to the issue of rater bias detection, the *l*_*z*_ statistic could be used to assess the likelihood of a rater’s assessment of an examinee given that examinee’s overall performance and the station specificity (difficulty and discrimination for items on the station-specific checklists).

In an attempt to further develop our repertoire of potential tools to detect rater bias, we conducted a Monte Carlo simulation study to investigate the power of the *l*_*z*_ PFS to detect biased raters. A simulation study makes it possible to control the rate of rater bias and, therefore, to estimate the detection rate of the statistic. This exploratory study aimed to establish the worthiness of pursuing the use of *l*_*z*_ to detect rater bias.

## Method

### Context

In this research, our primary goal was to test the detection of aberrant raters in a typical OSCE setup of 12 stations using the *l*_*z*_ PFS. The OSCE setup is for a relatively small sample of 48 candidates passing through all the stations, and we would need four iterations (cycles) to assess all 48 candidates on the same day. The raters are thought to be nested within the stations (remaining in the same station the entire day), and we stipulate that the OSCE psychometric properties (difficulty and discrimination of the checklist used for the stations) would be known to the administrators from prior administrations of the exam. In this section, the different choices we made for the simulation are further explained, starting with the use of the Monte Carlo simulation.

### Monte Carlo simulation

The Monte Carlo simulation methodology was applied in the context of this study as it allows for control of the design and the broad characteristics of the data generated by the simulation. Monte Carlo simulation is a methodology based on computational algorithms that rely on repeated random sampling. It is used in a large array of contexts but particularly when the probability of something occurring can be determined (e. g., to simulate radiation in dosimetry [39] or to analyze risk in finance [40]). For each data simulation aimed at understanding a statistical phenomenon, one result is obtained. Each independent result represents a possibility and is not of great interest on its own. When combined with results from other replications, a clearer picture emerges of the tendencies of the phenomenon of interest. In the context of RBA, we can use Monte Carlo simulations to generate data that represent RBA of examinee performance in a controlled setting to mimic different types of bias, meaning we can know exactly which rater was biased and to what degree. We can then establish whether the statistics used to detect them can do so successfully or not. Therein lies the elegance of Monte Carlo simulation studies.

In this research, the phenomenon of interest is the use of a PFS to detect raters who are biased, such as lenient or stringent raters. Different settings (e. g., raters with a more or less consistent rate of bias) are replicated multiple times, allowing us to gather information about the quality of detection according to the different manipulations. A Monte Carlo simulation can thus be used to draw conclusions about the power of a PFS to detect raters in a variety of OSCE contexts, something that would have been impossible with the use of empirical data.

### Design

We conducted a Monte Carlo simulation study with the primary outcome being the detection rates of a PFS, namely *l*_*z*_, while manipulating rater bias (stringent vs. lenient raters), number of biased raters (1, 2, 3, 4, 5 or 6 biased raters), and rate of bias per rater (10, 20, 30, 40, 50 or 60% bias per rater), to establish the appropriateness of using the *l*_*z*_ PFS to detect biased raters. We simulated a 12-station OSCE setting that could accommodate 48 students per day, where raters were nested in stations. Each station was assessed using a 10-item checklist. Each combination of factors (rater bias type $\times$ biased raters $\times$ bias rate) represents a ‘setting’, for a total of 72 simulated ‘settings’. Each setting was replicated 1,000 times. This simulation is akin to having an experimental design with 72 groups with 1,000 participants in each group. The data generation and the aforementioned factors are explained below.

#### *Data generation*:

We used the psychometric notation proposed by De Champlain [41]. The theta ability estimate is noted Θ, while difficulty and discrimination item parameters were designated a_i_ and b_i_, respectively, for the two-parameter logistic model (2PLM). Our decision to use the 2PLM was based on trying to find a balance between precision and parsimony. We conceptualize OSCE stations as having different difficulties and discriminations. Some authors could argue that the pseudo-guessing parameter (3PLM) could be applied to rater-based assessment data and as such would represent the chance of a rater attributing points to an examinee who did not show the said ability/knowledge. We adhere to the conceptualization initially proposed for pseudo-guessing as a parameter that applied in the context of multiple choice questions [42, 43]. In addition, using the 3PLM model implies substantially larger sample sizes compared with the 2PLM [43, 44]. Finally, the data available to us (Classical Test Theory difficulty and discrimination coefficients) only allowed us to model two parameters (a_i_ and b_i_).

#### Data distribution:

This simulation is based on OSCE station parameters from an HPE undergraduate program. More specifically, we used difficulty and discrimination coefficients for the 12 stations, and embedded 10-point checklists resulting from the administration of the OSCE. We used Laurencelle and Germain’s [45] transformation to go from Classical Test Theory item properties to the 2PLM item parameters. What can be gleaned from the checklist properties presented in Tab. 1 of the online Electronic Supplementary Material is that the simulated data would have an underlying negatively skewed data distribution since the stations were fairly easy—as is often observed in HPE. We simulated rater assessment patterns for cohorts of 48 examinees (levels of examinees ($\theta _{j})$ using a normal distribution while rater patterns were simulated using Bernoulli with probability $P_{i}(\theta _{j})$) having completed a 12-station OSCE, with each station being rated on a 10-item checklist (0 = fail, 1 = pass). Rater assessment vectors thus comprised a total of 480 checklist scores (i. e., the 10 scores per station given to the 48 examinees assessed).

**Table 1:** 10-Item Checklist properties used to simulate the data (Items difficuly (P_i_) and discrimination (D_i_))

		Stations											
		1	2	3	4	5	6	7	8	9	10	11	12
Item													
1	D_i_	0.43	0.67	0.39	0.89	0.92	0.82	0.77	0.67	0.67	0.82	0.59	0.95
	P_i_	−0.05	0.16	0.24	0.07	0.23	0.11	0.1	−0.35	0.31	0.09	0.11	0.14
2	D_i_	0.75	0.34	0.2	0.39	0.88	0.96	0.72	0.08	0.65	0.34	0.11	0.76
	P_i_	0.34	0.1	0.06	0.25	0.27	0.16	0.11	−0.26	−0.06	−0.01	0.3	0.13
3	D_i_	0.84	0.7	0.57	0.95	0.89	0.86	0.75	0.24	0.91	0.89	0.63	0.43
	P_i_	0.48	0.1	−0.03	0.12	0.24	0.12	0.44	−0.49	0.08	0.1	0.28	0.08
4	D_i_	0.97	0.28	0.48	0.56	0.97	0.72	0.97	0.01	0.45	0.62	0.21	0.73
	P_i_	0.07	−0.41	−0.09	0.55	0.3	0.14	0.14	−0.2	0.1	0.09	0.24	0.23
5	D_i_	0.18	0.8	0.57	0.7	0.97	0.75	0.93	0.92	0.84	0.13	0.78	0.94
	P_i_	−0.07	0.28	0.25	0.17	0.3	0.06	0.15	0.41	0.28	0.04	0.11	0.23
6	D_i_	0.18	0.69	0.67	0.93	0.93	0.71	0.72	0.86	0.53	0.81	0.31	0.82
	P_i_	−0.07	0.21	0.24	−0.03	0.16	0.17	0.29	0.22	0.43	0.33	0.36	0.22
7	D_i_	0.33	0.66	0.84	0.41	0.86	0.91	0.92	0.99	0.14	0.81	0.64	0.86
	P_i_	−0.06	0.41	0.34	0.09	0.07	0.1	0.28	−0.06	0.26	0.17	0.29	0.19
8	D_i_	0.34	0.03	0.87	0.52	0.65	0.83	0.68	0.92	0.22	0.62	0.11	0.95
	P_i_	−0.03	−0.12	0.29	0.62	0.29	0.19	0.18	0.34	0.24	0.06	0.22	0.38
9	D_i_	0.28	0.08	0.98	0.92	0.86	0.8	0.98	0.92	0.77	0.62	0.06	0.76
	P_i_	0.1	0.01	0.6	0.41	0.36	0.1	0.25	0.14	0.23	0.05	0.07	0.02
10	D_i_	0.3	0.07	0.9	0.61	0.67	0.57	0.78	0.95	0.69	0.6	0.1	0.05
	P_i_	−0.16	0.19	0.35	0.31	0.15	0.17	0.33	0.17	0.19	0.1	0.07	−0.19

#### Types of rater bias:

Two types of rater bias were simulated, that is, stringent raters and lenient raters. To achieve this, first rater vectors were randomly selected within a simulation, then items were randomly selected within a normal rater data vector. In the case of stringent raters, the randomly selected items were attributed a 0, while for lenient raters, they were attributed a 1. The number of rater vectors and items modified was determined by the rates described below.

#### Number of biased raters:

Six different numbers of biased raters per OSCE administration were simulated, that is 8% (*n* = 1) to 50% (*n* = 6) biased raters per exam. Although this latter bias level may seem high and unlikely, it was purposefully chosen to test the limit of the PFS studied.

#### Rate of bias per rater

Rate of bias represents the number of affected checklist items within a station for a given rater. Simulated bias rate varied from 10 to 60%. As suggested by McManus [28], a 10% bias rate reflects a systematically biased rater, which has little impact on the overall examinee score, while a 60% bias rate would have a greater impact on the examinee score. These bias rates reflect the maximum rates. While creating the biased rater data vectors, we randomly selected individual checklist items; as such, we might have selected ‘1’ when wanting to create a lenient rater, and ‘0’ when wanting to create a stringent rater, thus yielding a lower empirical bias rate.

### Analysis

#### l_z_ scores:

As a first step, the *l*_*z*_ *PFS.* An *l*_*z*_ score was computed for each rater, within each simulation. The* standardized likelihood l*_*z*_ index [36] was chosen for this study, as it is a standardized version of the *likelihood l*_*0*_ statistic [46] and could easily be adapted to our purpose, that is, the identification of rater bias. The *l*_*z*_ statistic represents the maximum value of the log-likelihood, which is an estimation of the probability logarithm of having a given answer pattern (in this case, it refers to the rater vector). More concretely, when applied to the context of a multiple choice question (MCQ) exam, the statistic will estimate the likelihood of an examinee’s answer to each question given 1) his performance on the other items and 2) other examinees’ performances. It then offers a measure of overall likelihood for the given examinee. This index has been shown to be able to detect spuriously high scores (akin to a lenient rater) and spuriously low scores (akin to a stringent rater). In summary, *l*_*z*_ establishes the likelihood of a vector of responses according to a given hypothetical model. In this case, we are testing the likelihood of rater response vectors according to the known checklist and station parameters and the examinees’ overall ability levels, both estimated using a 2PLM. When we parallel the situation to that of an MCQ exam, the *l*_*z*_ is thus used to compare a rater’s likelihood of their assessment of an examinee by 1) comparing their assessment of the examinee on the different checklist points, and 2) comparing their assessment of a given examinee to the other examinees in the group. The likelihood of their assessment pattern is established considering known values, such as the station’s item properties and the examinees ability level (score on the 12-station OSCE). The *l*_*z*_ statistic is thought to follow a normal distribution. As such, the value at the 95th percentile (for alpha 0.05) and the 99th percentile (for alpha 0.01) can be used as cut-off values. For more information on *l*_*z*_, its estimation and distribution, refer to Magis et al. [47].

#### Detection and false positive rates:

Raters that yield *l*_*z*_ scores greater than the cut-off values are identified as biased. Detection rates (the ratio between the number of raters correctly detected by the use of* l*_*z*_ and the total number of biased raters) and false-positive rates (the ratio between the number of raters incorrectly detected by the use of *l*_*z*_ and the total number of biased raters) were calculated for Type I error levels of 0.01 and 0.05. Those values are the most commonly used in Monte Carlo simulations [48, 49].

#### ANOVAs:

Data used for the ANOVAs were the resulting detection rates for each of the 1,000 replications for each of the 72 different simulated settings (2 levels of rater bias (stringent vs. lenient raters) $\times$ 6 levels for number of biased raters (1, 2, 3, 4, 5 or 6 biased raters) $\times$ 6 levels for rate of bias per rater (10, 20, 30, 40, 50 or 60% bias per rater)). In other words, the design resembles a situation where we would have 72 different groups with 1,000 participants in each group. We computed an ANOVA to establish whether the effects of the independent variables (*type of rater bias, number of biased raters, and rate of bias per rater*) were statistically significant*. *More specifically, ANOVAs were conducted to identify which factors (independent variables) might influence the detection rate of the *l*_*z*_ statistic (dependent variable). The partial eta squared ($\eta _{p}^{2}$) [46, 47] was used to estimate effect sizes, since the *F* statistic and the *p* value can be greatly influenced by the use of such large samples—achieved via the 1,000 replication of each simulated setting. As suggested by Olejnik and Algina [50], we used Cohen’s [51] thresholds of 0.01, 0.06, and 0.14 to establish small, medium, and large effects, respectively, between the variables. As is often the case in PFS studies, we only considered large effects [37, 52]*.*

Simulations were done using *R* [53] and, more specifically, [54] we used the Rirt package within R to simulate the original data and create biased raters. Descriptive statistics and ANOVAs were computed in SPSS 22.

## Results

**Fig. 1:**
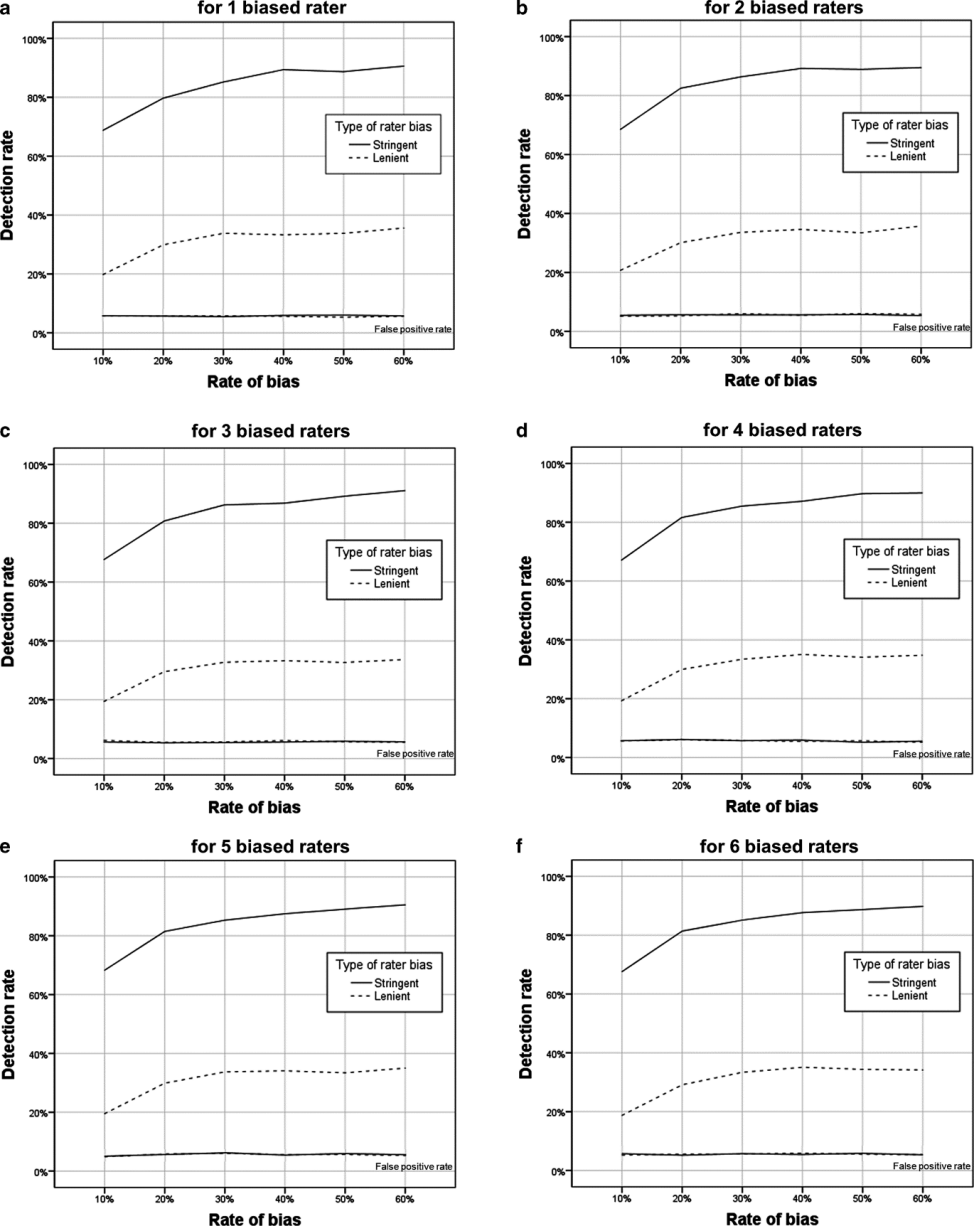
Detection rates per type of rater bias, rate of bias and number of biased raters

The detection rates and false-positive rates for *l*_*z*_ are presented in Fig. 1 of the online Electronic Supplementary Material per type of rater bias, rate of bias, and number of biased raters in a sample. What can be gleaned from Fig. 1 is that it was easier to detect stringent raters than lenient raters, independent of rate of bias and number of biased raters. The main effect of type of rater bias is corroborated by the ANOVA results. The overall mean detection rate for stringent raters was 0.84 (*SD* = 0.23), while it was 0.31 (*SD* = 0.28) for lenient raters (*F*(1,71998) = 80,788.37, *p* < 0.001, $\eta _{p}^{2}$ *=* 0.53).

It was also easier to detect biased raters with increased rates of bias, that is, raters were easier to detect when we had manipulated more answers within their completed checklist. This graphical interpretation is supported by the ANOVA results. The overall mean for the *l*_*z*_ detection rate was 0.63 (*SD* = 0.36) for a 60% rate of bias, and 0.44 (*SD* = 0.36) for a 10% rate of bias (*F*(5,71994) = 961.38, *p* < 0.001, $\eta _{p}^{2}$ = 0.06).

The number of biased raters in a sample did not influence the *l*_*z*_ detection rates (*F*(5,71994) = 80,788.37, *p* = 0.16, $\eta _{p}^{2}$ < 0.001). The interaction between type of biased rater and rate of bias did reach statistical significance (*F*(5,71994) = 534.63, *p* < 0.005, $\eta _{p}^{2}$ < 0.001). Since it did not have a large-effect size it is not discussed. All other interactions (number of biased raters × rater bias type, number of biased raters × type, number of biased raters × type of biased rater × rate of bias per rater) did not reach statistical significance.

The observed false-positive rates were close to the expected 0.05 for all conditions simulated, and were not influenced by the type of rater bias, rate of bias, or number of biased raters.

## Discussion

The use of performance-based assessment will continue to grow with the increased presence of competency-based education and, consequently, we will require that raters judge examinees’ performance. Given the extensive literature on rater limits, [11–15, 19–22, 25, 29, 31, 55–57] it is imperative that we rely on strategies that can help us to appropriately identify those raters who may need additional help or remediation, and cases where examinee scores need to be interpreted with caution. This study aimed to investigate if the *l*_*z*_ PFS can detect biased raters in the context of a simulated in-house performance-based assessment. The results of our study are promising. We observed detection rates as high as 90% for stringent raters, for whom we manipulated more than half their checklist. Less extreme conditions yielded lower detection rates. For example, detection rates for lenient raters with the minimum manipulation (10% rate of bias) hovered around 15%. Moreover, observed false-positive rates reflected the expected rates, suggesting that when raters are identified as aberrant, they most likely really are aberrant. The false-positive rates also act as a manipulation check that we are indeed identifying biased raters and not only noise or randomly identifying raters.

In addition to providing empirical evidence of the potential for the *l*_*z*_ PFS to detect biased raters, we have learned that for negatively skewed data, as is often the case in HPE, it is easier to detect stringent raters than lenient raters, and this was expected. The explanation for this observation is well documented in the broader PFS literature. We used item properties from a naturalistic context, that is, we used ‘real’ item properties from an assessment that was previously administered to examinees in an HPE program. As is often the case, we had a negatively skewed distribution in the data suggesting that examinees performed well. In a context of negatively skewed data, it is not surprising that more data points would have been modified to ‘0’ (stringent raters) than to ‘1’ (lenient raters) according to our manipulation protocol to simulate stringent and lenient raters. Research on PFS has often shown that it is easier to detect aberrant data vectors when more data are modified [33, 38, 46, 58, 59]. As such, when applying PFS to a naturalistic setting, in the presence of negatively skewed data, one should expect to more easily detect stringent raters than lenient raters, since they would deviate ‘more’ from the norm or expected patterns of response. It is thus not surprising that this study—similar to other studies on PFS—showed that more ‘aberrant’ (or deviant from a model) data vectors are easier to detect than less aberrant data vectors.

In addition, we used known item parameters in this study (stipulating that in an applied setting, we would use banked OSCE stations with known item parameters). In general, PFS aim to establish how much an observed response pattern deviates from the anticipated response pattern that can be predicted given an underlying measurement model (most often the item response theory). As such, the more a data vector, that is, the response pattern of a rater, strayed from its expected pattern, the easier it was to identify it as a biased rater. In the eventuality that PFS were applied in a naturalistic setting (real assessment data), where there is a need to estimate item parameters (no access to banked OSCE stations), the estimation of item parameters would be influenced by the ‘bias’ present in the data vectors and would make it more difficult to identify biased raters [52]. Moreover, the more bias present in a sample, the more difficult it would become to identify ‘deviant’ response vectors from a norm (which would itself be compromised by bias). While the magnitude of this effect has been investigated in the context of the traditional application of PFS, and it was found that many factors contribute to the specificity and sensitivity of the PFS, it remains to be investigated when PFS are applied to rater-based assessment.

Consequently, administrators wanting to detect biased raters must be aware that statistics such as the *l*_*z*_ PFS are influenced by the data distribution and, thus, the resulting detection rates may not be optimal. Nevertheless, the false-positive rates observed in this study give us great confidence that raters identified, even in the context of a skewed data distribution, are in fact biased and merit further investigation.

As is often the case with statistics, they bring to light, or hint at, something that merits further investigation. We have shown the *l*_*z*_ PFS to be able to detect both lenient and stringent raters in a controlled setting. However, in a real-life application, the *l*_*z*_ PFS would not be able to characterize the underlying bias on its own, meaning that an *l*_*z*_ cannot—in and of itself—indicate if the reason for the aberrance was stringency or leniency. An *l*_*z*_ score indicates that the observed assessment pattern deviates from the expected one. In a perspective of rater training and remediation, assessment administrators would have to use *l*_*z*_ scores in conjunction with their mean assessment scores to establish if the reason they were detected was because they were lenient or stringent. Fit statistics embedded in the MFRM can, however, be used to identify the underlying bias. Nonetheless, as previously mentioned, MFRM and the embedded fit statistics require that two distinct raters observe and score examinees’ performances, which renders their potential application in HPE unlikely. Future studies could investigate how PFS statistics applied to rater bias detection compare with the detection of rater bias using MFRM.

### Limits

Although the use of simulation studies allows us great control and precision when studying the appropriateness of statistical indices, it is not without limits. We used known item parameters to estimate the likelihood of raters’ assessment vectors (the scores they attributed to the examinees assessed). This decision most likely increased our detection rates. However, if we had used estimated item parameters, we would never have been able to differentiate rater properties from item/station properties. In other words, it would have been impossible to determine if the station was indeed difficult or if it was the rater who was stringent. Nonetheless, using known item properties supposes that one has access to banked OSCE stations, which may not be common practice, and thus greatly limits the potential applicability of the *l*_*z*_ index. In addition, and as is the case with Monte Carlo simulation studies, we generated a very clean portrait of bias in the sense that we only simulated one type of bias per group. This most likely does not reflect a natural occurrence of aberrant raters. Although we observed very interesting results that suggest that PFS could be used to detect aberrant raters, we cannot generalize these results to the use of PFS with estimated item/station parameters or real data. Finally, we focused on one rater bias, that is, leniency/stringency, and the results should not be generalized to other rater biases.

Before proceeding to studies with real data, future research should tackle the impact of cohort, other bias types, the scale used (checklist vs. global ratings), and the variability and estimation of item/station parameters. It would also be interesting to compare several PFS, as they may yield different results in different situations (e. g., Karabatsos [33], St-Onge et al. [37]), or even PFS with current detection strategies, such as MFRM. Once the application contexts for PFS have been well established, future studies should investigate the applicability of PFS to real data. With the increased use of workplace-based assessment, the problem of rater bias may persist. Future studies should investigate whether detection statistics can be applied to the identification of biased raters in that context.

The identification of biased raters is of extreme importance in the context of competency-based education, in which we see an increased reliance on rater-based assessment. With the *Standards *[60] voicing concern for the appropriateness of response processes, which includes how raters process data in the context of observing and scoring a performance, we need to explore strategies that would allow us to document the appropriateness of score interpretation in this regard. PFS seem to offer an interesting opportunity, although many questions still need to be answered before any large-scale implementation.
